# Placenta increta mimicking placental site trophoblastic tumor: A rare case report

**DOI:** 10.1016/j.ijscr.2024.110651

**Published:** 2024-11-26

**Authors:** Soheila Aminimoghaddam, Niloufar Sarchami, Elahe Ghaderi

**Affiliations:** a Department of Obstetrics and Gynecology, Iran University of Medical Sciences, Tehran, Iran; b Department of Obstetrics and Gynecology, School of Medicine, Iran University of Medical Sciences, Tehran, Iran

**Keywords:** Placenta, Trophoblastic, Tumor, Increta

## Abstract

**Introduction and importance:**

Placenta increta (PI) can mimic a rare malignant tumor called Placental-site trophoblastic tumor (PSTT) with similar laboratory and clinical findings. Histological findings can help correctly diagnose PI from rare malignant tumors of PSTT.

**Case presentation:**

According to our knowledge, only one case of PI has been reported in the literature, each of which had different characteristics, and various aspects of this lesion still need to be clarified. We reported a case of PI mimicking PSTT in a 37-year-old female. The uniqueness of the present case was its huge size and no history of abortion compared to the PI reported in the previous study.

**Clinical discussion:**

Our patients' ultrasound and immunohistochemical characteristics were similar to the case reported in the study. After the complete hysterectomy, the patient's bleeding stopped completely, and the patient remained disease-free during the two-year follow-up.

**Conclusion:**

PI can mimic a rare malignant tumor called PSTT with similar laboratory and clinical findings (Severe bleeding). The differential diagnosis of retained placenta in patients with extensive bleeding, either after vaginal delivery or after Cesarean section or abortion, should be carefully considered from PSTT. Both diseases (PSTT and PI) are treated with hysterectomy. Histological findings can help correctly diagnose PI from rare malignant tumors of PSTT.

## Highlights

• Placenta increta (PI) can mimic a rare malignant tumor called Placental-site trophoblastic tumor (PSTT) with similar laboratory and clinical findings.
• The uniqueness of the present case was its very large size (5 cm vs. 14 mm) and no history of abortion compared to the PI reported in the previous study.
• The differential diagnosis of retained placenta in patients with extensive bleeding, either after delivery or after Cesarean section or abortion, should be carefully considered from PSTT.
• Histological findings can help to correctly distinguish PI from the rare malignant tumor PSTT.
• Although ultrasound findings depend on the skill of the radiologist, however, ultrasound has the ability to distinguish arteriovenous malformations from PI and PSTT. While distinguishing PI from PSTT requires attention to the vascular pattern in MRI or CT scans findings.

## 1 Introduction

A placental-site trophoblastic tumor (PSTT) is a rare neoplastic transformation of trophoblastic cells involving implantation. It can occur after normal pregnancy, abortion, term delivery, ectopic pregnancy, or molar pregnancy. The pathological diagnosis of this lesion is challenging due to the uncertainty about the terminology, malignant potential, and prognostic factors, and it is considered one of the challenges of gynecologists [1]. Roughly 1–2 % of trophoblastic tumors form PSTT. In 1976, Korman et al., first described this distinct type of trophoblastic disease in 12 patients [2]. In 1981, Scully and Young named this lesion PSTT to represent the malignant potential of this tumor [3]. The neoplastic nature of this lesion was officially accepted in 183 by the World Health Organization (WHO), which introduced the term PSTT for this tumor [4]. In histological findings, mononuclear cells infiltrating the myometrium are this tumor's main distinguishing characteristic. They penetrate and multiply between the smooth muscle fibers and separate them through ropes and sheets. Cytological atypia may be seen in the findings. Another characteristic of this tumor is occasionally observing fibrinoid deposition [5].

The symptoms and manifestations of this lesion are extensive and can vary from a benign condition to an aggressive disease with a fatal outcome. Uterine perforation can occur in many cases [6,7] PSTT is a multifactorial disease whose etiology, epidemiology, and risk factors have not yet been fully determined [8]. However, hypotheses about the role of growth factor receptors and genetic disorders in its creation and spread have been proposed. Many studies have suggested that dysregulation of the epidermal growth factor receptor (EGF-R), disruption or inactivation of the p53 gene, and MIB-1 (Ki 67, a proliferation-associated antigen) may play a role in tumorigenesis and dissemination [8,9].

Diagnosis of PSTT is complex, and a case of PSTT may be misdiagnosed as the remaining parts of the placenta, PI/accreta, choriocarcinoma, static GTD, enlarged placental site, placental nodule, or epithelioid type of leiomyosarcoma [10]. Therefore, accurate diagnosis of this lesion is essential. Accurate diagnosis of high-risk and affected pregnancies enables optimal management.

Placenta increta (PI) is characterized by the placenta firmly adhering to the uterus and embedding itself in the muscular wall of the organ [11]. Complications in placental delivery and significant vaginal bleeding in the third trimester usually characterize PI. However, it can also present as post-curettage bleeding in the first and second trimesters. In many cases, patients may not show symptoms, and diagnosis is very difficult. Early diagnosis depends on awareness and accurately recognizing associated risk factors [11,12].

Due to the high overlap of their symptoms, differentiating between PT and PSTT can be challenging. MRI may help distinguish PI from PSTT [13]. The presence of arteriovenous abnormalities, including signal pits related to serpentine flow in the endometrium, uterine wall, and parametrium on T1- and T2-weighted MRI findings, may suggest PI. However, the presence of abnormalities may suggest PSTT and the presence or absence of a moderate T2-signal intensity mass in MRI and human chorionic gonadotropin is the only difference in diagnosis and differentiation. Arteriovenous malformations may exhibit a “cluster of grapes” because they contain large cystic spaces lacking a solid component [13,14].

The primary diagnostic tool for placenta accreta/ increta is ultrasound. When the ultrasound assessment is ambiguous, or for patients with high clinical risk factors, a computed tomography (CT) scan is used in diagnostic work to plan a cesarean delivery, and a Hysterectomy is used during childbirth.

This report presents a patient with a history of severe and multiple vaginal bleeding, finally diagnosed as PI, mimicking PSTT based on clinical, pathological, and MRI findings. The purpose of this case report is to introduce the challenges of distinguishing PI from PSTT and its management.

### 1.1 Informed consent statement

The patient provided written informed consent to use the data attributed to this case for publication.

The work has been reported in line with the SCARE criteria and the revised 2023 SCARE guidelines [15].

## 2 Case report

A 37-year-old woman G3P2, with a history of two uncomplicated c-sections, with no history of malignancies, 3 weeks before she was referred to our Gynecologic Oncology center, had been admitted to another gynecology center to manage her complaints of vaginal bleeding and terminating her early pregnancy failure which was shown in previous ultrasonography findings. Repeat ultrasound demonstrated a 13-week embryo with no growth and absence of cardiac activity in the lower segment of the uterus with extension to the cervix. The placenta was anterior, but analyzing the placenta accrete was impossible due to intracavitary clots. As the ultrasound findings were in favor of missed abortion, the patient was a candidate for pregnancy termination and given 800 μg misoprostol orally. Due to the moderate vaginal bleeding and incomplete abortion, the patient underwent curettage surgery.

Meanwhile the surgery, the patient encountered heavy vaginal bleeding, so uterine perforation was suspected. Therefore, a bedside ultrasound was performed. The ultrasound showed heterogeneous remnants of pregnancy, about 120 cc 14 × 38 mm in the lower segment of the uterus. The thickness of the myometrium at the site of the cesarean scar was thinner than normal, but the defect was not seen on the serous level of the uterus, and free fluid was not seen. Overall, the findings were not in favor of uterine dehiscence.

Meanwhile, suctioning the pregnancy remnants and controlling the bleeding, the patient had five units of hemoglobin drop. Therefore, two units of Pack Cells were transfused, and the patient was transferred to the ICU. A few days later, the patient was discharged. No evidence of gestational trophoblastic neoplasia (GTN) was mentioned in the final curettage pathology. Due to persistent vaginal bleeding, the patient returned to the same center one week after discharge. After re-evaluation of the patient, the new ultrasound revealed a large, ill-defined, hetero-echoic area with significant peripheral vascularity of about 52 × 45 × 58 mm and a volume of 72 cc in the distal cavity of the endometrium, with extension to the front of the myometrium at the site of the previous cesarean section. The differential diagnosis included: Retained product of conception and organized hematoma. Due to persistently low levels of serum B-HCG(about 70–80 mIU/ml) and persistent vaginal bleeding three weeks after surgery, the patient was referred to our tertiary hospital for further evaluation.

**Fig. 1:**
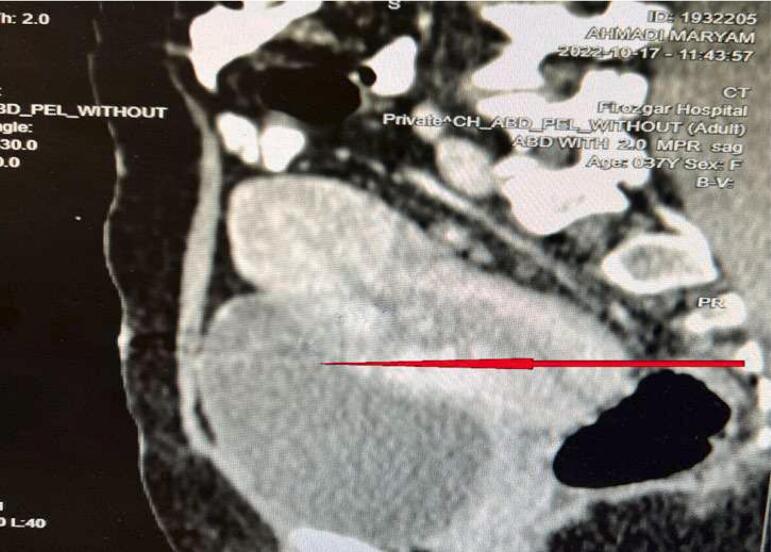
Coronal CT spiral view.

At the time of admission, the patient was evaluated clinically and radiographically. Color Doppler examination was performed to evaluate blood flow within the mass and the degree of vascularization. The color Doppler ultrasound showed a heterogeneous cystic mass of about 80 × 50 mm in the myometrium with a high degree of vascularization, representing Placental site trophoblastic tumor type 3 with AV-shunt. However, in the CT scan findings, there was a heterogeneous mass containing multiple internal tortuous vessels about 60 × 68 × 60 mm on the right side of the uterine body besides prominent parametrial vessels on both sides. According to the patient's history and recent curettage, the differential diagnoses of Placental Accreta, Increta, and Percreta were proposed for the patient. No clear fat plane was observed between the mass and the posterior wall of the bladder, suggesting the bladder wall was involved in the mass (Fig. 1). The patient was scheduled for laparotomy, and eventually, the patient underwent a hysterectomy. After a mallard surgical incision and releasing of omental adhesions to the abdominal wall, a 5 cm vascularized mass was observed in the isthmus region. The mass was completely attached and invaded to the bladder and the uterine serosa. Since complete dissection of mass from the bladder was impossible, after cystotomy and pushing the bladder down, a total hysterectomy was performed, and the sample was sent for pathological examination. Both ureters were checked, and bilateral pelvic lymphadenectomy, up to the iliac level, was performed. In the end, the bladder was repaired in two layers.

**Fig. 2:**
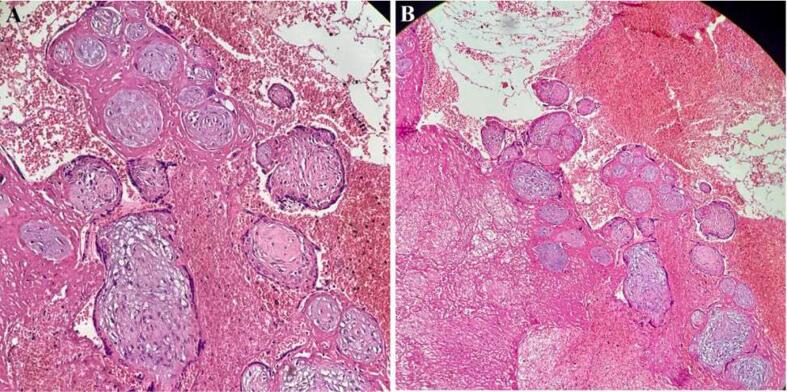
(A) A well-differentiated necrotic mass measuring 4 × 4× 3 cm in the lower part of the uterus that penetrated the myometrium(X40*10).(B) Chorionic villi and trophoblasts observed in the background of necrosis and hemorrhage in the microscopic examination of formalin-preserved paraffin-embedded sections.

Final pathologic findings revealed a round, well-defined brown-creamy mass of about 4*4*3 cm was identified in the lower uterine segment. On cut sections, hemorrhagic areas are noted. On sectioning chorionic villi, they are identified and extended into the thinned myometrium, and some necrotic changes are compatible with the diagnosis of placenta increta. Microscopic examination of formalin-preserved paraffin-embedded sections showed chorionic villi and trophoblasts in the background of necrosis and hemorrhage (Fig. 2). Moreover, all the pelvic lymph nodes were negative for malignancy.

After the surgery, the beta-hcg level decreased rapidly, and the patient was discharged in very good health. She was followed up, and at 24 months, she is still disease-free without any complications.

## 3 Discussion

Since PI mimicking PSTT is a rare lesion, several aspects of this lesion, including early diagnosis and treatment management, are still one of the challenges of gynecological surgery [4,16,17]. Only one case of PI mimicking PSTT has been reported [16]. Although limited cases of placenta mimicking have been reported, to our knowledge, only one case has been reported.

Remained PI can be associated with serious complications and catastrophic postpartum hemorrhage [18]. Studies have shown that persistent remains are associated with an increased risk of maternal and neonatal outcomes, including postpartum hemorrhage, hysterectomy transfusion, preterm delivery, and the need for neonatal intensive care unit admission, which places a heavy burden on health systems [18,19]. Therefore, timely diagnosis and management are crucial. However, diagnosis is very difficult due to the overlapping symptoms and findings on imaging. A review of 14 cases suspected of PI revealed a hyperintense region on T2-weighted images with temporary early enhancement and a lack of significant delayed enhancement, which indicates PI [13].

In 2021, M Clark et al., [16] reported a case of PI mimicking PSTT in a 35-year-old pregnant woman at 8 weeks' gestation. As in our case, the patient had no previous surgery or comorbidities. This patient had a history of two uncomplicated vaginal deliveries and two abortions. As in our study, the patient had presented with bleeding. Gonadotropin (HCG) level was 65,083 IU/L. In the follow-up of 10 weeks gestational age, ultrasound findings of the fetus's lack of growth and cardiac activity (abortion) were confirmed. A mass containing dilated serpiginous vessels measuring up to 14 mm was observed. The mass seen in our case was much larger than those reported in the study of M Clark et al., and our patient, unlike those reported in their study, had no history of previous abortion. Our patients' ultrasound and immunohistochemical profile was similar to that reported by M Clark et al. In this study, as in our study, the final diagnosis of PI was based on histopathological findings. These findings suggest that abnormal vascular patterns and severe bleeding may suggest PI.

H Zhang et al., [19] showed that the diagnostic accuracy of ultrasound and MRI for PI was very low, and the misdiagnosis based on ultrasound and MRI was high. PI was finally diagnosed based on the hCG level and histopathological findings. U Fidan et al., [20], in 2016, reported a 37-year-old pregnant woman (gravida 2, parity 1) with abnormal vaginal bleeding who had a history of two pregnancies (one successful, uncomplicated pregnancy and one abortion at the 15th week of pregnancy due to rupture of membranes). Dilation-curettage was performed for the remains of the abortion. The patient returned three weeks after the curettage due to continued vaginal bleeding. As in our case, Doppler ultrasound evaluation showed a heterogeneous solid mass, 5.5 × 6 cm in diameter, with a high-velocity peripheral vascular pattern in the uterine cavity. As in our study, a hysterectomy was performed. The final diagnosis of placenta accreta mimicking PSTT was confirmed in the final histopathological evaluation of the mass. They reported in their case that placenta accreta can mimic the rare malignant tumor of PSTT, which has the same laboratory and clinical findings. These reports show that with the increase in cesarean rate [16,[20], [21], [22]] more abnormalities in placental attachment will be reported by obstetricians and gynecologists, which highlights the importance of paying attention to the diagnosis of placenta accreta and increta in patients with extensive bleeding, either after abortion or after delivery by cesarean or even natural delivery, especially when it is determined that hysterectomy It is known to treat both conditions (PSTT and placenta accreta/increta) and both conditions can be associated with heavy bleeding.

In another study, 2023, C Berriozabal et al., [23] reported a placenta accreta mimicking PSTT in a 31-year-old woman (Gravida 6, para 2, oboration4) with a history of two caesareans and three curettages. During the control ultrasound scan after the last spontaneous abortion, a uterine mass was observed. MRI showed a heterogeneous uterus, a mass measuring 63 × 43 × 42 mm, containing many abnormal vascular structures penetrating the myometrial wall, suggesting PSTT. In this case, like our case, a hysterectomy was performed to control the bleeding.

Special attention should be paid to the differential diagnosis, including PSTT, other uterine tumors, and placenta accreta/increta after vaginal abortion in women with a history of cesarean section.

Early and accurate diagnosis and correct intervention can prevent maternal mortality and even help preserve the uterus for women who want to get pregnant.

Our study had limitations that may reduce its generalizability to other cases and should be noted. The lack of access to diagnostic tools, including MRI, at the primary care center where the patient was referred made the diagnosis and management of the patient challenging. Also, the presence of blood and clots prevents accurate examination of the increase with ultrasound, thus increasing the rate of misdiagnosis.

## 4 Conclusion

PI can mimic a rare malignant tumor called PSTT with similar laboratory and clinical findings (heavy bleeding). The differential diagnosis of retained placenta in patients with massive bleeding, whether postpartum or after cesarean section or abortion, should be carefully considered from PSTT. Both diseases (PSTT and PI) are treated with hysterectomy. Histological findings can help distinguish PI from rare malignant tumor PSTT correctly. Although ultrasound findings depend on the radiologist's skill, ultrasound can distinguish arteriovenous malformations from PI and PSTT. Distinguishing PI from PSTT requires attention to the vascular pattern in MRI or CT scan findings.

## Research registration

N/A.

## CRediT authorship contribution statement

Soheila Aminimoghaddama: Analysis and interpretation of data, drafting the article, final approval of the version to be submitted.

Niloufar Sarchami: Analysis and interpretation of data, drafting the article, final approval of the version to be submitted.

Elahe Ghaderib: Analysis and interpretation of data, drafting the article, final approval of the version to be submitted.

## Consent

Written informed consent was obtained from the patient for publication of this case report and accompanying images. A copy of the written consent is available for review by the Editor-in-Chief of this journal on request.

## Ethical approval

The ethics committee of Iran University of medical science approved this study.

## Guarantor

Dr. Niloufar Sarchami

## Provenance and peer review

Not commissioned, externally peer-reviewed.

## Sources of funding

N/A.

## Declaration of competing interest

The authors declare that they have no competing interests.
